# Supplementary material for: Identification of photoperiod-induced specific miRNAs in the adrenal glands of Sunite sheep (Ovis aries)
Source: Front Vet Sci. 2022 Jul 22;9:888207. doi: 10.3389/fvets.2022.888207 (PMC9354845; doi:10.3389/fvets.2022.888207)
Supplement: Supplementary file 1 [file Data_Sheet_1.ZIP › Supplementary file/miRDeep2 file.zip!/miRDeep2 file/Know_Structure/SP42A3/oar-mir-544.pdf]

miRBase precursor : oar-mir-544
Total read count : 325
oar-miR-544-5p read count : 309
oar-miR-544-3p read count : 16
remaining reads : 0

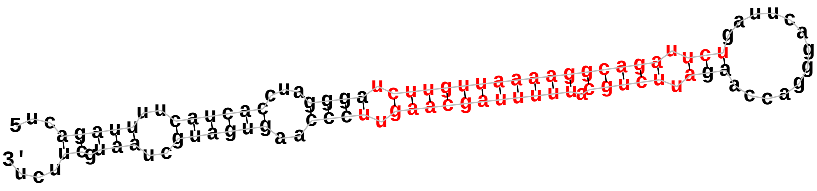

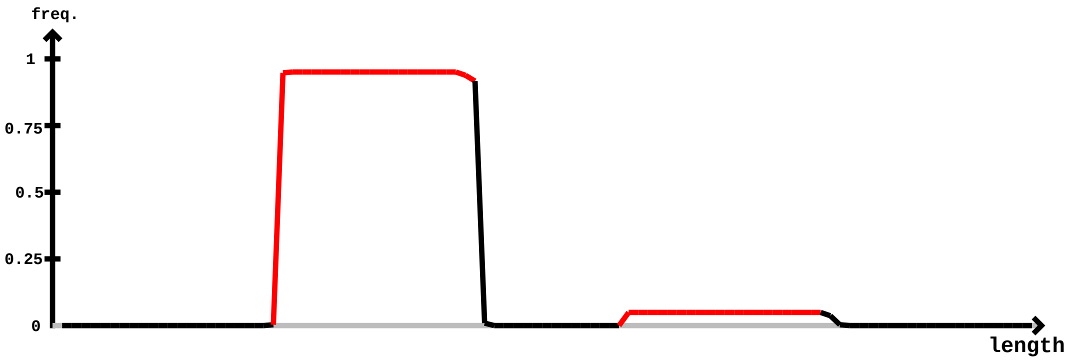


```
                          oar-miR-544-5p
                                               oar-miR-544-3p
5'- ucagauuuucaucaccuagggaucuuguuaaaaggcagauucugauucagggaccaagauucugcauuuuuagcaaguucccaagugaugcuaaugcuucu   -3'   exp
    ..(((((..((((((...((((.((((((((((((((((.(((.............))).))))).))))))))))).))))..))))))..))).))...   reads   mm   sample
    .....................aucuuguuaaaaggcagauu............................................................   1       0    seq
    ......................ucuuguuaaaaggcagauu............................................................   1       0    seq
    ......................ucuuguuaaaaggcagauA............................................................   2       1    seq
    ......................ucuuguuaaaaggcagauuc...........................................................   7       0    seq
    ......................ucuuguuaaaaggcagauucA..........................................................   22      1    seq
    ......................Ccuuguuaaaaggcagauucu..........................................................   3       1    seq
    ......................ucuuAuuaaaaggcagauucu..........................................................   1       1    seq
    ......................ucuuguuaaaaAgcagauucu..........................................................   2       1    seq
    ......................ucuuguuaaaaggcagauucu..........................................................   263     0    seq
    ......................ucuuguuaaaaggUagauucu..........................................................   1       1    seq
    ......................ucuuguuaGaaggcagauucu..........................................................   2       1    seq
    ......................ucuuguuaaaaggcagauucuA.........................................................   2       1    seq
    ......................ucuuguuaaaaggcagauucuU.........................................................   1       1    seq
    .......................cuuguuaaaaggcagauucu..........................................................   1       0    seq
    ..........................................................auucugcauuuuuagcaaguu......................   4       0    seq
    ..........................................................auucugcauuuuuagcaaguuU.....................   4       1    seq
    ..........................................................auucugcauuuuuagcaaguuc.....................   7       0    seq
    ..........................................................auucugcauuuuuagcaaguucc....................   1       0    seq
```
